# Antimicrobial use and resistance in food animal production: food safety and associated concerns in Sub-Saharan Africa

**DOI:** 10.1007/s10123-023-00462-x

**Published:** 2023-12-06

**Authors:** Timothy Obiebe Jason Odey, Williams Omotola Tanimowo, Kayode Olayinka Afolabi, Iqbal Kabir Jahid, Rine Christopher Reuben

**Affiliations:** 1 Department of Biological Sciences, Faculty of Natural, Applied, and Health Sciences, Anchor University, Lagos, Nigeria; 2 Pathogenic Yeasts Research Group, Department of Microbiology and Biochemistry, University of The Free State, Bloemfontein, South Africa; 3 Department of Microbiology, Jashore University of Science and Technology, Jashore, 7408 Bangladesh; 4 Area of Biochemistry and Molecular Biology, OneHealth-UR Research Group, University of La Rioja, 26006 Logroño, Spain

**Keywords:** Antimicrobial resistance, Antimicrobial use, Food safety, Food animals, Sub-Saharan Africa

## Abstract

The use of antimicrobials in food animal (FA) production is a common practice all over the world, with even greater usage and dependence in the developing world, including Sub-Saharan Africa (SSA). However, this practice which serves obvious economic benefits to producers has raised public health concerns over the last decades, thus driving the selection and dissemination of antimicrobial resistance and adversely impacting food safety and environmental health. This review presents the current and comprehensive antimicrobial usage practices in food animal production across SSA. We further highlighted the overall regional drivers as well as the public health, environmental, and economic impact of antimicrobial use in the production of food animals. Antimicrobial use is likely to increase with even exacerbated outcomes unless cost-effective, safe, and sustainable alternatives to antibiotics, especially probiotics, prebiotics, bacteriocins, antimicrobial peptides, bacteriophages, vaccines, etc. are urgently advocated for and used in food animal production in SSA. These, in addition to the implementation of strong legislation on antimicrobial use, and improved hygiene will help mitigate the public health concerns associated with antimicrobial use in food animals and improve the well-being and safety of food animals and their products.

## Introduction

The use of antimicrobials extends beyond their known clinical application to combat and cure diseases in humans. They have also been employed in the agricultural industry to treat diseases in animals and prevent the occurrence of diseases, and in sub-therapeutic doses in feeds, as growth-promoting agents, enhancing feed conversion for high mass and product yield (Manyi-Loh et al. 2018). Their use in animal production extends well over 6 decades, dating back to the mid-1940s when increased production of antibiotics for the treatment of ailing soldiers from the Second World War was initiated. This afforded the availability and accessibility of lyophilized drugs for use in the veterinary world, which yielded promising results (Van et al. 2019).

In the USA alone, the estimated consumption of antibiotics in animal production accounts for about 80% of the total fraction of antibiotics produced (VanBoeckel et al. 2015) of which about 63% include antibiotics that are employed for treatment in humans (Van et al. 2019). Globally, the average annual consumption of antibiotics in animal production is estimated at 172 mg/kg, 148 mg/kg, and 45 mg/kg for pigs, poultry, and cattle, respectively, with a projected rise of 67% (i.e., from about 63,000 tons to about 106,000 tons) between 2010 and 2030 (VanBoeckel et al. 2015). A relatively recent study also provided supporting data, putting the global average annual antibiotics consumption at 192 mg/PCU, 68 mg/PCU, and 43 mg/PCU for pigs, chickens, and cattle, respectively, with a projected increase from 93,000 tons in 2017 to 103,000 tons in 2030, signifying 11.5% increase (Tiseo et al. 2020).

Following this trend of use, these antibiotics which are otherwise essential for the continuous production of healthy animals supplied into the food chain now acclaim significant interest. This is because the class, the reason for use, and the mechanism of action of these antimicrobials utilized in animal production are similar, and in most cases the same as those employed for therapeutic purposes in humans (Manyi-Loh et al. 2018). More concerns are currently building up as the veterinary use of antibiotics in animal production has been associated with the emergence of multidrug-resistant pathogens in humans. Some studies have demonstrated a correlation between the occurrence and subsequent rise in the frequency of virulence and antibiotic resistance determinants in zoonotic bacteria due to antibiotic use in animal production (Capita and Alonso-Calleja 2013; Mdegela et al. 2021). Antimicrobial resistance (AMR) has turned the center of the One Health Initiative, being intertwined into the agricultural, environmental, and public health sectors. Thus, it requires a global synergetic approach from these sectors to effectively control the menace. This challenge is particularly glaring in developing and resource-limited countries, including Sub-Saharan Africa (SSA). Genotypic studies around this region provide evidence of high transmission of AMR across livestock, the environment, and the human population, due to frequent unsanitary interactions between food animals and humans as a result of poor living conditions (Caudell et al. 2020).

In Africa, there is widespread indiscriminate use of antibiotics, poor clinical care, inadequate regulations on antibiotics, and a lack of regional surveillance on AMR and antimicrobial use (AMU) (Lim et al. 2021; Craig et al. 2022; Gulumbe et al. 2022). No significant progress has been achieved in the implementation of antibiotic stewardship and surveillance programs in human and animal systems. According to the recent WHO report on AMR surveillance, only 23 African countries have enrolled in the Global Antimicrobial Resistance and Use Surveillance System (GLASS) (WHO 2021a). Out of the 23 countries, only 15 have successfully reported annual data on the antibiotic surveillance program in 2021. Despite the increasing AMR crisis in Africa, the implementation of the AMR guidelines has been inadequate in most countries. The implementation of the guidelines is necessary for ensuring stewardship in antimicrobial use and can reduce the development and spread of antimicrobial resistance (WHO 2021b). We have been assessing the level of AMU and also characterizing the spread of AMR among major pathogens of public health importance within the human, animal, and food systems. In developing sustainable solutions to mitigating AMU and AMR, we have in recent years evaluated alternatives to antibiotics including probiotics, bacteriocins, and postbiotics for broad applications in the One Health continuum (Reuben et al. 2019, 2020, 2021a, 2021b, 2022; Al-Emran et al. 2022).

Apart from the emergence of resistance among microorganisms, another concern due to the abusive use of antibiotics in FAs is the dysbiosis of the animals’ microbiome and the presence of drug residue in both animal wastes and processed animal products. These drug residues adversely affect the environment and instigate food safety concerns like toxicity, sensitization, allergies, and carcinogenicity, following product consumption (Manyi-Loh et al. 2018).

The lack of comprehensive data on AMU and AMR in SSA is tied to the paucity of requisite information, due to inadequate and inefficient data collection and surveillance systems (Manyi-Loh et al. 2018; Kimera et al. 2020b). This review presents the current and comprehensive antimicrobial usage practices in food animal production across SSA, especially the specific antibiotics and antibiotic classes commonly used, animal species, and reasons for use (i.e., therapeutic, prophylaxis, and growth promotion). Additionally, we highlighted the overall regional drivers of antibiotic use in food animal production as well as the public health, environmental, and economic impacts in SSA. Our review serves as a regional atlas of antimicrobial use in food animals and a valuable resource for future research, development, and application of sustainable measures to reduce the adverse impacts of antimicrobial use and resistance in food animals thus improving food safety.

## Literature search and data extraction

In February 2023, we performed an exhaustive literature search in the Web of Science database (www.webofscience.com) to assess the use of antibiotics in animal production in Sub-Saharan Africa. Our keyword search consisted of the terms “antibiotics” OR “antimicrobials” OR “antibiotic use” OR “antimicrobial use” OR “antibiotic resistance” OR “antimicrobial resistance” OR “animal production” OR “food safety”, “food animals” OR “Africa” OR “Sub-Saharan Africa.” Furthermore, additional search was conducted in other sources such as PubMed and Google Scholar. We assessed articles that were published in the English language after carefully and independently reviewing their titles and abstracts. The selected articles were thoroughly assessed to extract relevant information regarding antimicrobial use and resistance.

## Therapeutic and prophylactic use of antimicrobials in food animal production in SSA

**Fig. 1:**
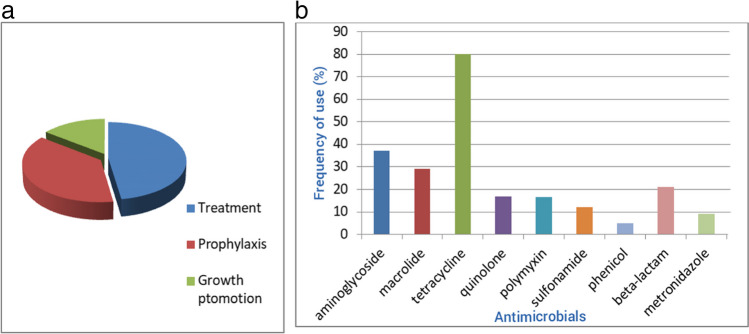
**a** Purpose of antimicrobial use in FAs within Sub-Saharan Africa. **b** Frequency of use of different antimicrobials employed in animal production in Sub-Saharan Africa

The use of antimicrobials in intensive animal and food production systems aimed at maintaining animal health and optimum production is of great necessity (Mshana et al. 2021). Their use has reduced the cost of food animal production, allowing the rearing of FAs in small and confined spaces, and increasing profit margin (Mankhomwa et al. 2022). However, undesirable outcomes through the course of use, such as the selection and spread of AMR, raise major concerns (Mshana et al. 2021). These fears arise as a higher integration of sub-therapeutic dosing in raising FAs, in terms of prophylaxis, is becoming a common practice in developing countries, especially in Sub-Saharan Africa (SSA), thus implicating great consequences of AMR in animals and humans alike (Ayukekbong et al. 2017) (Figs. 1a, b).

## Therapeutic use of antimicrobials

The primary use of antimicrobials therapeutically is directed at treatment, where sick animals are administered specific antimicrobials for the elimination of the pathogen(s) causing the disease (Aarestrup 2015). For instance, an estimated 15% of cattle in North America receive treatment for bovine respiratory disease with fluoroquinolones, annually (Cameron and McAllister 2016). The central concern in the therapeutic use of antimicrobials lies in the abuse or misuse of these agents, as prudent use of antimicrobials optimizes therapeutic efficacy and reduces pressure for selection of resistance and concerns of toxicity in FAs and their products (Capita and Alonso-Calleja 2013; Mankhomwa et al. 2022).

In a study in Ogun state, southwestern Nigeria, 72.3% of the pig farmers admitted using antibiotics for the treatment of sick pigs. The most reported antibiotics utilized for this purpose were tetracycline, tylosin, and gentamycin (Adebowale et al. 2020). In a similar research in Abeokuta, Nigeria, 50% of live bird sellers treated their sick birds with antibiotics. Tetracycline, chloramphenicol, and metronidazole were the most mentioned antimicrobials used on the birds (Adebowale et al. 2022). In both studies, proper guidelines for antimicrobial use in animal production were poorly practiced by the farmers. Furthermore, about 80% and 72% of the study population, i.e., pig farmers and live bird sellers, respectively, reportedly purchased the drugs over the counter and administered them to the animals at their discretion. Little or no withdrawal was observed among the farmers concerning AMU in FAs (Adebowale et al. 2020, 2022). Compared with the previous studies, increased (97%) use of antibiotics for treatment was reported among livestock (cattle, sheep, goats, and poultry) farmers in Oyo and Kaduna states, Nigeria (Ojo et al. 2016). The study also revealed tetracycline, tylosin, ciprofloxacin, chloramphenicol, gentamycin, and colistin to be the most consumed antimicrobials in livestock production. In the same vein, Adesokan et al. (2015) reported tetracycline (33.6%), fluoroquinolones (26.5%), and aminoglycosides/beta-lactams (20.4%) as the topmost antimicrobials used in animal production in Nigeria. Between 2010 and 2012, the consumption of antimicrobials as reported in the study increased by 40.4%, an unmatched increase when compared to an annual livestock growth rate of 2% in the country (Adesokan et al. 2015). A continuous pattern in the class of antimicrobials consumed in FA production across West Africa was maintained. In Dormaa district, Ghana, all (100%) the participants in a study reportedly used antimicrobials for the treatment of their poultry birds, with most farmers administering tetracycline usually in combination with tylosin, chloramphenicol, neomycin sulfate, or furaltadone (Johnson et al. 2017). Again, the estimated antibiotic use for the treatment of poultry birds was 25% and 64% among commercial and domestic farmers, respectively, in the Ashanti region of Ghana (Paintsil et al. 2021). Tetracycline accounted for the highest percentage of antibiotics used (62.5%), followed by neomycin (56.2%), tylosin (40.6%), streptomycin (28.1%), and colistin (25%) (Paintsil et al. 2021).

Emphatically, the use of antimicrobials for treatment appears to follow a consistent trend across the eastern and central regions of Africa. In Ethiopia, about 84% of livestock farmers used antibiotics for the treatment of sick animals (cattle, sheep, goats, equines, and poultry). Tetracycline, aminoglycosides, and sulfonamide-trimethoprim were the most frequently administered antibiotics by the farmers (Gemeda et al. 2020). Conversely, a subsequent study within the Oromia zone, Ethiopia, revealed a lower use (41.5% ) of antibiotics for the treatment of poultry, pigs, goats, and cattle (Gebeyehu et al. 2021). Supporting data for the antimicrobial class used as reported in a study in Tanzania revealed a 14% use of antimicrobials for the treatment of poultry, pigs, and ruminants. Tetracycline, penicillin, sulfonamides, macrolides, and quinolones were the implicated drugs utilized during treatment (Mdegela et al. 2021). However, a contradictory report was presented from another study in Tanzania, with a higher (80.5%) use of antibiotics for treatment in poultry and pigs. Tetracycline, quinolones, and sulfonamides, among other antibiotics were commonly administered to treat diarrhea, typhoid, and respiratory diseases in animals (Kimera et al. 2020a). Furthermore, the use of human antibiotics in animal treatment, accompanied by poor knowledge of the prevention and control of diseases, was notably linked to the educational status of the farmers (Kimera et al. 2020a). Aligning with this report, a study in Kiambu district, Kenya, found a 90% use of antibiotics for the treatment of sick poultry birds. Tetracycline dominated use with tylosin, erythromycin, colistin, and neomycin following in decreasing order of use (Kiambi et al. 2021). Likewise, another study among Maasai pastoralists in Kenya reported high use of tetracycline (65%) with penicillin-streptomycin (28%) and azithromycin (7%) following in descending order. However, a higher practice of self-medication was reported among the population when compared to other studies, as the Maasai pastoralists considered themselves “doctors and custodians of veterinary drug knowledge” (Mangesho et al. 2021). In a study among livestock farmers in Rwanda, all (100%) of the respondents admitted the use of antibiotics for the treatment of sick animals. Penicillin and streptomycin were the most used antibiotics commonly referred to as “peni-streptomycin” (Manishimwe et al. 2017). Equally important, studies in Cameroon highlighted the high use of tetracycline, beta-lactams, sulfonamides, last-resort quinolones, and colistin in FA production, especially for pigs and poultry (Mickecz et al. 2020; Moffo et al. 2020).

Furthermore, research across southern Africa confirms the widespread use of tetracycline for treatment in FAs (Caudell et al. 2020; Mupfunya et al. 2020; Mankhomwa et al. 2022). In Blantyre, Malawi, tetracycline was more frequently used for the treatment of poultry, pigs, and goats, with streptomycin and erythromycin following next. The use of colistin, a critical antibiotic for human medicine, was also reported to be employed for the treatment of sick animals (Mankhomwa et al. 2022). About 84 and 90% of farmers used antibiotics for the treatment of sick animals in Zambia and Zimbabwe, respectively. Conversely, high prudence in AMU and farm practices was reported from the poultry farmers in both countries as compared to most others within the SSA region (Caudell et al. 2020).

## Prophylactic use of antimicrobials

Prophylaxis therapy in FAs practically involves the administration of antimicrobials to prevent the occurrence of disease in an animal perceived to be at risk, though lacking clinical symptoms of the disease (Aarestrup 2015). A similar concept, metaphylaxis involves treating a larger group of animals having members both with and without clinical symptoms of the disease. For example, in many countries including North America, asymptomatic calves are administered macrolides for prophylaxis or metaphylaxis of bovine respiratory disease (BRD) (Cameron and McAllister 2016). Widespread across low- and middle-income countries, with specific interests in the SSA region, is the prophylactic use of antibiotics in animal production (Moffo et al. 2020; Mdegela et al. 2021; Adebowale et al. 2022; Bedekelabou et al. 2022). Enforcing this practice is the increasing demand for animal protein from a rapidly growing population (Mankhomwa et al. 2022). Evidence from current research suggests that more antibiotics are administered to FAs for prophylaxis, than any other reason as it reduces the costs of production and mortality rates (Kimera et al. 2020b; Mdegela et al. 2021; Paintsil et al. 2021; Mankhomwa et al. 2022).

In Nigeria, a study carried out among pig farmers in Ogun state revealed that 36.9% of the study population used antibiotics for prophylaxis. Tetracycline, gentamycin, and tylosin were the most frequently used antibiotics (Adebowale et al. 2020). In a similar study conducted in 2021 among live bird sellers in Abeokuta, southwestern Nigeria, 20% of the respondents reportedly administered antibiotics to their birds for prophylaxis. The predominant antimicrobials used were tetracycline and chloramphenicol. High use of metronidazole was also reported (Adebowale et al. 2022). In a retrospective study, Adesokan et al. (2015) reported tetracycline (33.6%), fluoroquinolones (26.5%), and aminoglycosides/beta-lactams (20.4%) as the topmost antimicrobials used in animal production in Nigeria. Conversely, in Northwestern Nigeria, a high level of resistance was documented against sulfonamides (65%), tetracycline (58%), and ciprofloxacin (46%), although this was not necessarily in correspondence with their use in the farm animals (Jibril et al. 2021). The report of antimicrobials used in poultry in Oyo and Kaduna states, Nigeria, presents an array of different antimicrobials which included tetracycline, tylosin, ciprofloxacin, chloramphenicol, gentamycin, and colistin as the most frequently used drugs. Over 87% of farmers utilized antibiotics in animal production for prophylaxis in Oyo and Kaduna states (Ojo et al. 2016).

In a study in Ghana, tetracycline (62.5%) accounted for the largest percentage of antibiotics used among poultry farmers in the Dormaa district; this was frequently sold in rebranded forms usually in combination with other agents like tylosin, chloramphenicol, neomycin sulfate, furaltadone, and vitamins (Johnson et al. 2017). Consequentially, tetracycline residues were determined to be 24.3%, 21.7%, and 12.2% at mean concentrations of 0.02 ± 0.003, 0.02 ± 001, and 0.01 ± 0.008 in μg/g in eggs, kidneys, and liver samples from the birds reared within the Dormaa district. Although the highlighted concentrations were below the acceptable maximum limit for the drug, there is a possibility that it will increase with time as 86% of farmers recruited in the study administered antibiotics to their poultry birds for prophylaxis (Johnson et al. 2017). In a more recent study by Paintsil et al. (2021) within the Ashanti region of Ghana, 97% and 43% of commercial and domestic poultry farmers, respectively, used antibiotics in animal production. However, when compared to Johnson et al. (2017), a greater fraction of commercial and domestic farmers in Ashanti used antibiotics more often for prophylaxis (94% and 66%) (Paintsil et al. 2021). Findings from a similar study in southern Togo showed that poultry farmers (93%) utilized antibiotics in production more than pig farmers (50%). About 84% of the farmers who consented to antimicrobial use utilized them as prophylaxis in the production of poultry and pigs (Bedekelabou et al. 2022). Almost all the farmers (98%) recruited in the study reportedly failed to consult a veterinarian for proper clinical and laboratory diagnosis as well as antibiotic prescriptions. Conforming to the trend across West Africa, the most frequently used antimicrobials based on the study were tetracycline (85%), erythromycin/tylosin (73%), and colistin (57%). Poor biosecurity practices were also widely observed, especially in pig farms (Bedekelabou et al. 2022).

Notably, a relatively unchanging trend in antimicrobial use was observed across the central and eastern regions of Sub-Saharan Africa. A cross-sectional study among livestock owners in Ethiopia showed a 10% use of antibiotics among pastoralists as prophylaxis (Gemeda et al. 2020). This falls below the result from another study in Ethiopia by Gebeyehu et al. (2021), with a reported 39.1% of farmers admitting antibiotics use for both prophylaxis and metaphylaxis in poultry, cattle, sheep, and goats. In addition, the farmers included in the study displayed poor knowledge of AMR and AMU in FAs. Contrary to the common trend across West African countries was the use of anthelmintics as prophylaxis (>64%). The most frequently used antibiotics included tetracycline, aminoglycosides, and sulfonamide-trimethoprim (Gemeda et al. 2020).

In Tanzania, a multi-method survey was used to investigate the use of antimicrobials in livestock (poultry, pigs, and ruminants) production. This survey reported a 60% use of antimicrobials for prophylaxis (Mdegela et al. 2021). The use of tetracycline, penicillin, sulfonamides, macrolides, and quinolones was commonly reported among the farmers. The farmers usually stabilize (administer prophylaxis) animals ready for sale with antibiotics, without taking cognizance of the stipulated withdrawal periods. In addition to this, it was observed that prohibited drugs such as furazolidones are being used among poultry farmers, and were also available in some veterinary stores (Mdegela et al. 2021). In the same vein, higher use of tetracycline, quinolones, and sulfonamides, among other antibiotics, was also observed in a cross-sectional study in the eastern region of Tanzania (Kimera et al. 2020a). Essentially, the respondents (farmers) within eastern Tanzania admitted a substantial use of antimicrobials for prophylaxis in pigs and poultry (87.6%) (Kimera et al. 2020a). High dependence and application of tetracycline were also reported among poultry farmers in Kiambu district, Kenya. Other antimicrobials administered to poultry birds include tylosin, erythromycin, colistin, and neomycin. About 75% of the farmers administered antibiotics to the poultry birds as prophylaxis (Kiambi et al. 2021). Comparably, in a study among the Maasai pastoralists in Kenya, tetracycline (65%) was the predominantly used antibiotic for their livestock, followed by penicillin-streptomycin (28%) and azithromycin (7%), supporting the findings from other regions within Sub-Saharan Africa (Mangesho et al. 2021).

As per the trend across central and eastern Africa, about 44% use of antibiotics for prophylaxis was reported in cattle, sheep, goats, pigs, and poultry production by the farmers surveyed in a study in Rwanda (Manishimwe et al. 2017). The use of antibiotics as reported in the study was strongly correlated to location, as farmers in urban areas displayed a more prudent use of antibiotics than those in rural areas. The most commonly used antibiotic among the surveyed farmers was “peni-streptomycin” (Manishimwe et al. 2017). Reports from Mickecz et al. (2020) however contradicted the trend earlier observed in the entire central African region, as antimicrobial use among farmers in Uganda was shown to be relatively lower (35%), with 7% AMU in poultry production. This could be explained based on the population studied, as the investigation focused more on domestic and small-scale farmers rather than commercial animal production systems as observed in other studies, establishing a clear connection between antibiotics use and the size of the farm.

In Cameroon, a 6-year study to determine the patterns of importation of antibiotics and how it relates to animal production in the country was performed. The findings showed that tetracycline (31.71%), sulfonamide (23.84%), beta-lactams (10.17%), and quinolones (11.11%) were the most commonly imported antibiotics (Moffo et al. 2020). There was an observed 104% increase in the number of active substances utilized in FAs over the study period, with higher mean values of antibiotics used in pigs and poultry than in cattle and other ruminants. A critical finding was the use of extremely important and last-resort antibiotics against multidrug-resistant pathogens in humans, for animal production, especially pigs and poultry (Moffo et al. 2020). Also, in another study in Cameroon, high usage of tetracycline (43.7%) in poultry, followed by amoxicillin (12.8%), colistin (11.9%), and enrofloxacin (8.8%), was reported. In the study, 64.1% of the respondents utilized antibiotics for prophylaxis (Moffo et al. 2020). Major concerns were raised as regards the reported use of antibiotics in raising day-old chicks (15.6%), combined use of antibiotics and diuretics (48.8%), and tetracycline administration alongside minerals in poultry birds. All these practices intended for prophylaxis ensue sub-lethal concentrations of antibiotics in animals, thereby triggering stress in bacteria and consequently increasing the chances of mutation resulting in drug resistance (Van-der-Horst et al. 2013; Moffo et al. 2020).

In the southern African region, the common use of tetracycline was found among farmers in South Africa, with 72% observing withdrawal periods after the treatment course (Mupfunya et al. 2020). Antibiotics were sourced at the local veterinary clinic (60%) and agricultural retail stores (34%). In a qualitative study in Blantyre, Malawi, tetracycline was more frequently used by the farmers, followed by streptomycin and erythromycin (Mankhomwa et al. 2022). It was also reported that human antibiotics are being used in the treatment of animals, as the cost of veterinary drugs is much more expensive than their human alternatives. Although colistin has been banned in Malawi, they are still being dispensed, with veterinary officers claiming ignorance of such regulations (Mankhomwa et al. 2022). The summary of therapeutic and prophylactic uses of antibiotics in FAs within Sub-Saharan Africa is highlighted in Table 1.

**Table 1:** Therapeutic and prophylactic use of antibiotics in food animals within Sub-Saharan Africa

Antibiotic class	Antibiotic	Animal species	Percentage (%)	Country	Reference
Therapeutic use					
Tetracycline, aminoglycoside and macrolide	Tetracycline, gentamycin, and tylosin	Pigs	73.2	Nigeria	Adebowale et al. (2020)
Tetracycline, phenicol, nitroimidazole	Tetracycline, chloramphenicol, metronidazole	Poultry	50.0	Nigeria	Adebowale et al. (2022)
Tetracycline, polymyxin, fluoroquinolone, macrolide, aminoglycoside, sulfonamide	Oxytetracycline/doxycycline, colistin, ciprofloxacin, erythromycin, tylosin, streptomycin, neomycin, sulfonamide	Poultry	75.5 and 42.7	Nigeria	Alhaji et al. (2018)
Tetracycline, macrolide, fluoroquinolone, phenicol, aminoglycoside, polymyxins	Tetracycline, tylosin, ciprofloxacin chloramphenicol gentamycin, and colistin	Poultry	94.0	Nigeria	Ojo et al. (2016)
Tetracycline, macrolide, phenicol, aminoglycoside	Tetracycline, tylosin, chloramphenicol, neomycin	Poultry	100.0	Ghana	Johnson et al. (2017)
Tetracycline, aminoglycoside, macrolide, polymyxin	Tetracycline, neomycin, streptomycin, tylosin, colistin	Poultry	25.0 and 64.0	Ghana	Paintsil et al. (2021)
Tetracycline, aminoglycoside, macrolide, polymyxin.	Tetracycline, erythromycin tylosin, colistin.	Pigs and poultry	N/P	Togo	Bedekelabou et al. (2022)
N/P	N/P	Poultry, sheep, goats, cattle	41.5	Ethiopia	Gebeyehu et al. (2021)
Tetracyclines, aminoglycosides, sulfonamide-trimethoprim	N/P	Cattle, sheep, goats, equines, poultry	84.0	Ethiopia	Gemeda et al. (2020)
Tetracyclines, penicillins, sulfonamides, macrolides, and quinolones	Oxytetracycline, cloxacillin, sulfamethoxazole, tylosin, norfloxacin	Poultry, pigs, ruminants (cattle, sheep, goats)	14.0	Tanzania	Mdegela et al. (2021)
Tetracyclines, quinolones, sulfonamides, macrolides, and aminoglycosides	Oxytetracycline, tetracycline, enrofloxacin, sulfadiazine trimethoprim, tylosin, neomycin	Pigs and poultry	80.5	Tanzania	Kimera et al. (2020a)
Tetracyclines, macrolide, aminoglycoside, polymyxin	Tetracycline, tylosin, erythromycin, neomycin, colistin	Poultry	90.0	Kenya	Kiambi et al. (2021)
Tetracyclines, beta-lactams, macrolide	Tetracycline, penicillin, azithromycin	Cattle, sheep, goats	N/P	Kenya	Mangesho et al. (2021)
Beta-lactams, aminoglycoside	Penicillin, streptomycin	Poultry, pigs, ruminants (cattle, sheep, goats)	100.0	Rwanda	Manishimwe et al. (2017)
N/P	N/P	Poultry, pigs ruminants (cattle, sheep, goats)	58.0	Uganda	Mickecz et al. (2020)
Tetracycline, sulfonamides, quinolones, beta-lactams	Oxytetracycline, doxycycline, sulfadiazine, ciprofloxacin, enrofloxacin, amoxicillin, ampicillin	Pigs, poultry, ruminants, cattle	N/P	Cameroon	Moffo et al. (2020)
Tetracycline, beta-lactam, polymyxin, fluoroquinolone	Tetracycline, amoxicillin, colistin, enrofloxacin	Poultry	N/P	Cameroon	Moffo et al. (2020)
Tetracycline	Terramycin	Cattle	N/P	South Africa	Mupfunya et al. (2020)
Tetracycline, aminoglycoside, macrolide polymyxin	Tetracyclines, streptomycin erythromycin, colistin	Poultry, goats, pigs	N/P	Malawi	Mankhomwa et al. (2022)
Prophylactic use					
Tetracycline, aminoglycoside, and macrolide	Tetracycline, gentamycin, and tylosin	Pigs	32.9	Nigeria	Adebowale et al. (2020)
Tetracycline, phenicol, nitroimidazole	Tetracycline, chloramphenicol, metronidazole	Poultry	20.0	Nigeria	Adebowale et al. (2022)
Tetracycline, polymyxin, fluoroquinolone, macrolide, aminoglycoside, sulfonamide	Oxytetracycline/doxycycline, colistin, ciprofloxacin, erythromycin, tylosin, streptomycin, neomycin, sulfonamide	Poultry	N/P	Nigeria	Alhaji et al. (2018)
Tetracycline, macrolide, fluoroquinolone, phenicol, aminoglycoside, polymyxins	Tetracycline, tylosin, ciprofloxacin chloramphenicol gentamycin, and colistin.	Poultry	87.0	Nigeria	Ojo et al. (2016)
Tetracycline, macrolide, phenicol, aminoglycoside	Tetracycline, tylosin, chloramphenicol, neomycin	Poultry	84.0	Ghana	Johnson et al. (2017)
Tetracycline, aminoglycoside, macrolide, polymyxin	Tetracycline, neomycin, streptomycin, tylosin, colistin	Poultry	94.0 and 66.0	Ghana	Paintsil et al. (2021)
Tetracycline, aminoglycoside, macrolide, polymyxin.	Tetracycline, erythromycin tylosin, colistin.	Pigs and poultry	84.0	Togo	Bedekelabou et al. (2022)
N/P	N/P	Poultry, sheep, goats, cattle	38.1	Ethiopia	Gebeyehu et al. (2021)
Tetracyclines, aminoglycosides, sulfonamide-trimethoprim	N/P	Cattle, sheep, goats, equines, poultry	10.0.	Ethiopia	Gemeda et al. (2020)
Tetracyclines, penicillins, sulfonamides, macrolides, and quinolones	Oxytetracycline, cloxacillin, sulfamethoxazole, tylosin, norfloxacin	Poultry, pigs, ruminants (cattle, sheep, goats)	60.0	Tanzania	Mdegela et al. (2021)
Tetracyclines, quinolones, sulfonamides, penicillins, macrolides, and aminoglycosides	Oxytetracycline, tetracycline, enrofloxacin, sulfadiazine trimethoprim, tylosin, neomycin	Pigs and poultry	87.6	Tanzania	Kimera et al. (2020a)
Tetracyclines, macrolide, aminoglycoside, polymyxin	Tetracycline, tylosin, erythromycin, neomycin, colistin	Poultry	75.0	Kenya	Kiambi et al. (2021)
Tetracyclines, beta-lactams, macrolide.	Tetracycline, penicillin, azithromycin	Cattle, sheep, goats	N/P	Kenya	Mangesho et al. (2021)
Beta-lactams, aminoglycoside	Penicillin, streptomycin	Poultry, pigs, ruminants (cattle, sheep, goats)	44.0	Rwanda	Manishimwe et al. (2017)
N/P	N/P	Poultry, pigs ruminants (cattle, sheep, goats)	44.0	Uganda	Mickecz et al. (2020)
Tetracycline, sulfonamides, quinolones, beta-lactams	Oxytetracycline, doxycycline, sulfadiazine, ciprofloxacin, enrofloxacin, amoxicillin, ampicillin	Pigs, poultry, ruminants, cattle	N/P	Cameroon	Moffo et al. (2020)
Tetracycline, beta-lactam, polymyxin, fluoroquinolone	Tetracycline, amoxicillin, colistin, enrofloxacin	Poultry	64.1	Cameroon	Moffo et al. (2020)
Tetracycline	Terramycin	Cattle	N/P	South Africa	Mupfunya et al. (2020)
Tetracycline, aminoglycoside, macrolide polymyxin	Tetracyclines, streptomycin erythromycin, colistin	Poultry, goats, pigs	N/P	Malawi	Mankhomwa et al. (2022)

*N/P* not provide

## Antimicrobials use as growth promoters in food animal production in SSA

Emerging reports indicate that more antibiotics are consumed in animal production systems than in human medicine (Manishimwe et al. 2017; Van et al. 2019; Mankhomwa et al. 2022). Of the antimicrobials consumed by FAs, 37% have no equivalent use in the treatment of human infections as most of this estimated fraction include ionophores; therefore, concerns about the risks of transfer of resistance arising from their use are abated (Cameron and McAllister 2016; Argudin et al. 2017). Nevertheless, the larger fraction (~63%) of these antimicrobials constitutes antibiotics utilized for human therapeutics (Cameron and McAllister 2016). Considerable concerns erupt with the overuse of antibiotics including aminoglycosides, third-generation cephalosporins, polymyxins, and macrolides which are considered “critically important” antibiotics in food animal production (Van et al. 2019; Kiambi et al. 2021; Mdegela et al. 2021). Unlike in therapeutic dosing, for growth promotion, antimicrobials are usually added to animal feeds in sub-lethal amounts, consequently evoking resistance in bacteria by continuous selective pressure (Cogliani et al. 2011; Van et al. 2019). Since the observation that chlortetracycline residues of *Streptomyces aerofaciens* increased growth rate in farm animals fed with dried mycelia in the 1940s, the growth-promoting potential of antibiotics has been explored in animal production (Castanon 2007; Van et al. 2019). The improved growth rate observed in animals can be attributed to interactions of the antibiotic(s) with the microflora of the animal’s gut, as it cuts down the competitive uptake of nutrients by the gut microflora, reduces opportunistic pathogens, and abates the production of growth repression metabolites by the microflora. Growth is also improved by the thinning of the intestinal villi and walls, thereby enhancing digestibility and nutrient uptake (Capita and Alonso-Calleja 2013; Reuben et al. 2021a). The application of antibiotics to animal feeds for growth promotion became more pronounced in the 1950s and 1960s when different antibiotics with different mechanisms of action were incorporated into animal feeds. In-fed supplementation of animals with antibiotics continued until the emergence of public health concerns attributed to drug residues in meat and animal products, increased AMR, gut dysbiosis, etc. Due to these concerns, the European Union (EU) and the UK in 2006 banned the in-fed use of antimicrobials as growth promoters in animal husbandry (European Commission 2005). Similarly, the USA, in a less strict policy, proscribed the use of medically important antibiotics as growth promoters in food animal production (FDA 2012). However, the contrary is the case with most developing countries, especially SSA where there is weak and/or no legislation for antibiotic use in FAs. Bringing SSA into focus, the extensive use of antimicrobials in food animal production goes on amply unregulated. This can be underpinned by an increased population growth rate, propping a higher demand for animal protein and tilting animal production to an industrial scale. Consequently, antimicrobial use in the production process increases continuously (Obimakinde et al. 2017; Tiseo et al. 2020).

In southwestern Nigeria, a report by Adebowale et al. (2020) showed significant use of antibiotics on pigs by the farmers for growth promotion (29.2%) (Table 2). Tetracycline, gentamycin, and tylosin were reported to be the most used antibiotics among the farmers. A similar study among poultry farmers in north central Nigeria revealed the same trend in the purpose and use of antibiotics. In-fed antibiotics for growth promotion were estimated at 10.5 and 7.3% for poultry farmers and local bird keepers in Nigeria (Alhaji et al. 2018). In Ashanti, Ghana, AMU for growth promotion was determined to be at 35 and 11% in poultry as revealed by a study (Paintsil et al. 2021). More commercial poultry farms consumed antibiotics than backyard or domestic farms, an observed variance that was attributed to the size of the farm and the need for growth promotion among other reasons in commercial poultries. The commercial farmers being more profit-oriented administered copious amounts of antibiotics to poultry birds to boost growth, decrease mortality, and increase feed conversion rate (Paintsil et al. 2021).

**Table 2:** In-fed antibiotics for growth promotion in food animals within Sub-Saharan Africa

Antibiotic class	Antibiotic	Animal species	Percentage used (%)	Effect	Country	Reference
Tetracycline, aminoglycoside and macrolide	Tetracycline, gentamycin, and tylosin	Pigs	29.2	Growth promotion	Nigeria	Adebowale et al. (2020)
Tetracycline, polymyxin, fluoroquinolone, macrolide, aminoglycoside	Oxytetracycline/doxycycline, colistin, ciprofloxacin, erythromycin, tylosin, streptomycin, neomycin.	Poultry	10.5 and 7.3	Growth promotion	Nigeria	Alhaji et al. (2018)
Tetracycline, aminoglycoside, macrolide, polymyxin	Tetracycline, neomycin, streptomycin, tylosin, colistin	Poultry	35.0 and 11.0	Increased animal growth	Ghana	Paintsil et al. (2021)
Tetracycline, macrolide, aminoglycoside	Tetracycline, tylosin, streptomycin	Poultry, cattle, goats	8.0	Increased animal growth	Ghana	Caudell et al. (2020)
Tetracycline, macrolide, aminoglycoside	Tetracycline, tylosin, streptomycin	Poultry, cattle, goats	7.0 and 10.0	Increased animal growth	Kenya	Caudell et al. (2020)
Tetracycline, aminoglycoside, macrolide	Tetracycline, tylosin, streptomycin	Poultry, cattle, goats	40.0 and 60.0	Increased animal growth	Tanzania	Caudell et al. (2020)
Tetracycline, macrolide, aminoglycoside	Tetracycline, tylosin, streptomycin	Poultry, cattle, goats	10.0 and 15.0	Increased animal growth	Zambia	Caudell et al. (2020)
Tetracycline, macrolide, aminoglycoside	Tetracycline, tylosin, streptomycin	Poultry, cattle, goats	3.0	Increased animal growth	Zimbabwe	Caudell et al. (2020)
Tetracyclines, aminoglycosides, sulfonamide-trimethoprim, Anthelmintics*	N/P	Cattle, sheep, goats, equines, poultry	3.0 and 40.0*	Livestock fattening	Ethiopia	Gemeda et al. (2020)
N/P	N/P	Poultry, sheep, goats, cattle	19.4	Increased animal growth	Ethiopia	Gebeyehu et al. (2021)
Tetracyclines, penicillins, sulfonamides, macrolides, and quinolones	Oxytetracycline, cloxacillin, sulfamethoxazole, tylosin, norfloxacin	Poultry, pigs, ruminants (cattle, sheep, goats)	26.0	Growth promotion	Tanzania	Mdegela et al. (2021)
Tetracyclines, macrolide, aminoglycoside, polymyxin	Tetracyclines, tylosin, erythromycin, neomycin, colistin	Poultry	24.3	Increased egg production, Increased feed conversion	Kenya	Kiambi et al. (2021)
N/P	N/P	Poultry, pigs ruminants (cattle, sheep, goats)	6.0 and 3.0	Growth promotion	Uganda	Mickecz et al. (2020)
Tetracycline, beta-lactam, polymyxin, fluoroquinolone	Tetracycline, amoxicillin, colistin, enrofloxacin	Poultry	82.3	Growth promotion	Cameroon	Moffo et al. (2020)
Tetracycline, aminoglycoside, macrolide polymyxin	Tetracyclines, streptomycin erythromycin, colistin	Poultry, goats, pigs	N/P	Increased animal growth	Malawi	Mankhomwa et al. (2022)

*GP* growth promotion, *N/P* not provided, *anthelmintics used for growth promotion

A study in Kenya reported the use of special drugs consisting chiefly of oxytetracycline, neomycin, and vitamins as growth promoter formulations, with manufacturers including details of egg production promotion, growth-boosting, and vitamin supplements during stress and disease state, reduced mortality, and increased conversion rate, on the drug indication of the packaging material. An estimated 24.3% of the farmers in the study admitted the administration of these special formulations for growth promotion to their FAs (Kiambi et al. 2021). In a study involving five SSA states (Ghana, Kenya, Tanzania, Zambia, and Zimbabwe), the farmers (predominantly pastoralists) in Tanzania reported a high use of antibiotics for growth promotion in food animals. While about 60% of the farmers in Tanzania incorporated antibiotics for growth promotion in their livestock, less than 20% of the farmers from the four other countries had this practice (Caudell et al. 2020). The markedly high use of in-fed antibiotics for growth promotion carefully aligns with an observed 50% increment in treatment failure experienced by Tanzanian pastoralists since they began farming (Caudell et al. 2020). Keeping with the trend in other studies across the eastern region of SSA, about 19.4% of Ethiopian farmers used antibiotics for growth promotion in cattle, sheep, goats, and poultry, a lower percentage as compared to what was obtained in Tanzania. Antimicrobial prudence varied significantly with knowledge of AMU and biosecurity as well as the level of education of the farmers (Gebeyehu et al. 2021). A similar study among livestock keepers (majorly pastoralists) in Ethiopia revealed a relatively low use of antibiotics as growth promoters (< 4%). However, a high fraction of the surveyed farmers utilized anthelmintic as a growth-promoting agent (~40%) (Gemeda et al. 2020). Inappropriate use of antibiotics was reported to be high among the pastoralists, aligning with the report of Caudell et al. (2020) from eastern SSA (Gemeda et al. 2020). In consonance with other studies in the eastern SSA region, about 26% of Tanzanian farmers used antibiotics for growth promotion purposes (Table 2), with tetracycline, colistin, and neomycin constituting the main antibiotics incorporated into feed formulations (Mdegela et al. 2021). A widespread practice within Tanzania was the use of neomycin-oxytetracycline preparation in water for chicks. Poor regulations surrounded feed formulations and production within this region, as private individuals designed and formulated their feeds introducing antibiotics and coccidiostats based on their discretion. This level of drug misuse was also accompanied by high levels of drug residue in animal products, as biosecurity measures and withdrawal periods were poorly adhered to by the farmers (Mdegela et al. 2021).

There was an extensive use of antibiotics in Rwanda, with most of the surveyed farmers (97.4%) admitting the use of antibiotics in poultry, pigs, and ruminant production. About 26.5% of the respondents administered antibiotics to promote animal growth. The autonomous use (no prescription) of antibiotics was also reported to be common by 55.6% of the local respondents (Manishimwe et al. 2017). Mickecz et al. (2020) in their study showed antibiotics use increased with the size of the flock this ranged from 96 to 63% in Uganda. Antibiotics use for growth promotion was relatively below par as observed by the trend across the SSA region; this ranged from 6% in ruminants to 3% in cattle. The use of amoxicillin, oxytetracycline, and colistin as growth promoters was observed by poultry farmers in Cameroon (Moffo et al. 2020). About 83.3% of the surveyed farmers administered in-fed antibiotics for growth promotion in FAs. This was the highest measured response for growth promoter use of antibiotics by researchers across the SSA region (Moffo et al. 2020). The antibiotics were also combined with diuretics and administered to day-old chicks as earlier mentioned. In the study, knowledge of AMU and AMR was positively correlated with AMU and biosecurity practices among farmers (Moffo et al. 2020).

In the southern region of SSA, a qualitative study reported high use of tetracycline, which was usually sold in combination with vitamins and other drugs such as erythromycin and colistin. As earlier mentioned, this was done despite the ban on colistin use in FAs in Malawi (Mankhomwa et al. 2022). The use of “vitamin mix” to boost animal production was also common. These mixes contained low doses of multiple antibiotics (Mankhomwa et al. 2022). Figure 1 outlines the frequency, purposes, and antibiotics mostly use in animal production in in SSA.

## The culpability of food animals in the emergence and spread of antimicrobial resistance in SSA

**Fig. 2:**
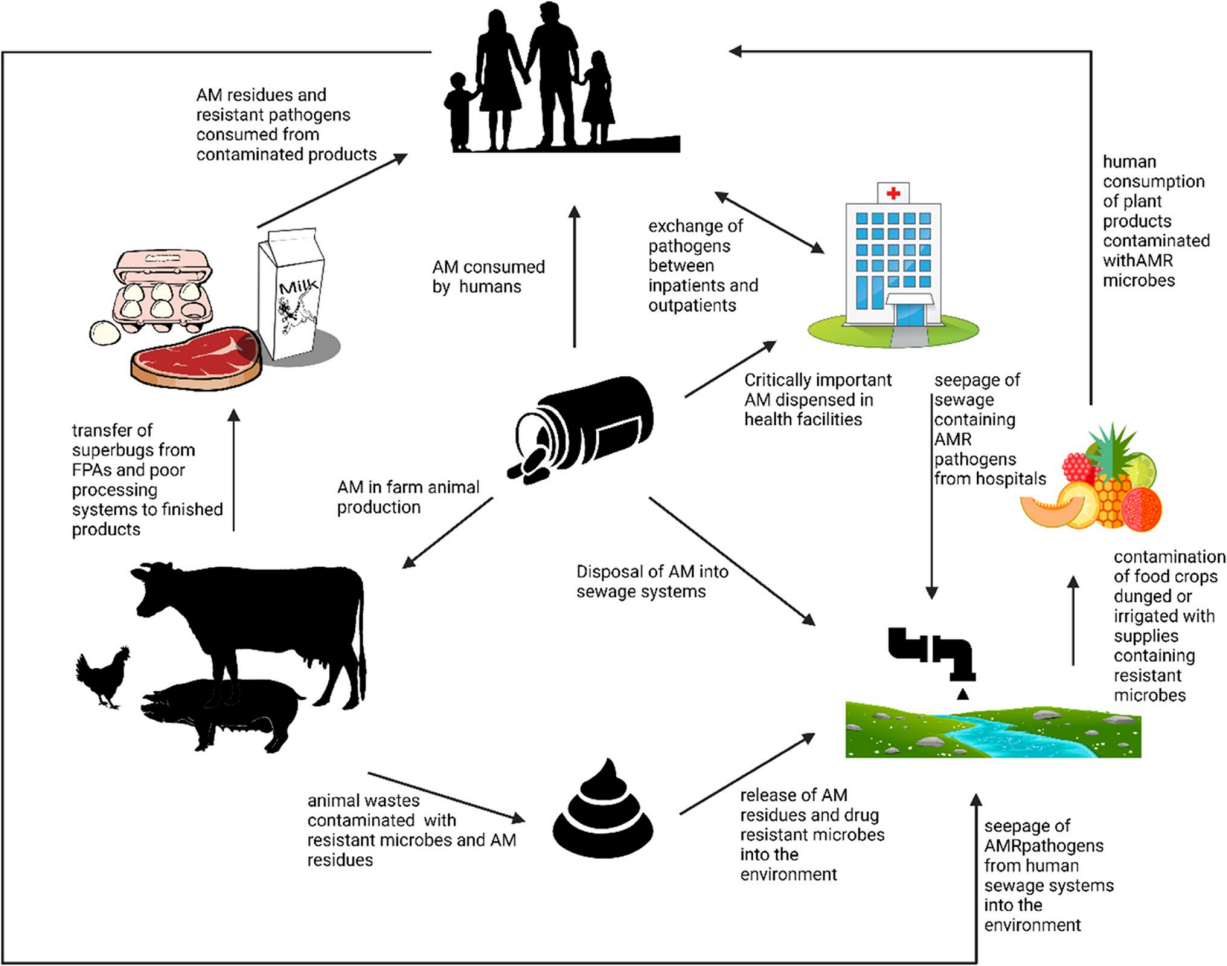
Complex interactions of antimicrobial use and resistance among humans, animals, and the environment. AM, antimicrobial

Human and animal health has always been threatened by the emergence (or reemergence) and continuous spread of AMR. The unabated and widespread use of antibiotics in food animal production in SSA has further exacerbated the public health burden associated with AMR within the region (Ma et al. 2021). Due to the lack of robust or poorly implemented public health laws regulating antibiotic use, the indiscriminate use of antimicrobials has become endemic, thus promoting requisite conditions that facilitate the spread of AMR within the food systems. This practice has further implicated FAs as major reservoirs for the circulation, maintenance, and spread of AMR pathogens within the animal-human-environment (food) continuum (Ma et al. 2021; Wolfe 2023). The spread of AMR has been established to occur through direct contact with the animals or their wastes, via contaminated food or animal products, or indirectly through the environment (Fig. 2) (Xu et al. 2022). The person-to-person transmission within the human population then sets in after a successful animal-to-human zoonotic transmission (Caudell et al. 2020). To a large extent, it is believed that the emergence and spread of AMR in humans and the environment are aggravated and sustained through the indiscriminate use of antimicrobials in FAs (Guardabassi 2004; Caudell et al. 2020). Furthermore, advances in technology especially the ease of trade and transport of animal and food products across the globe further increase the complicity of food animals in the emergence and spread of AMR thereby constituting a major threat to global health (Guardabassi 2004; Kimera et al. 2020b).

The development of antibiotic-resistant traits in bacteria typically involves a sequential process of evolution and horizontal gene transfer, oftentimes spanning a prolonged period (Fitzgerald 2019). However, bacteria evolution within the context of AMR in the last few decades has seen its most rapid turnaround. This process of evolution and dissemination of resistance has been accelerated by the poor use of antimicrobials for veterinary and animal husbandry purposes. This enhances the use of horizontal gene transfer by bacterial species for the acquisition of different functional genes including antibiotic resistance (Van et al. 2019). Similarly, the intensive use of antimicrobials has increased the occurrence of AMR in human pathogens, thereby increasing the severity of disease outcomes (Rasmussen et al. 2015). Several (pathogenic) bacteria are shared between animals and humans, and the environment (foods). On exposure to stress factors such as antibiotics, alterations in pH, or temperature, susceptible species die off leaving behind the resistant ones. As a result of the selective pressure, a resistant progeny arises that could withstand, multiply, and survive the stress factors. With time, the resistant population gradually replaces the susceptible ones, and their spread continues. For instance, the prolonged use of antibiotics (low doses and sub-lethal exposure) where potency is inadequate greatly enhances the chances of bacteria to adapt, tolerate and thrive in the presence of the antibiotic(s) rather than being eliminated (Capita and Alonso-Calleja 2013; Rasmussen et al. 2015). Sequel to the selective pressure, mobile genetic elements, such as plasmids, may be picked up by susceptible bacteria, leading to the transmission of genes encoding resistance between phylogenetically related and even unrelated bacteria (Ma et al. 2014; Fitzgerald 2019).

Greater concerns come in as different genes encoding resistance to different antibiotics are usually positioned on the same mobile genetic material including transposons, plasmids, and integrons. Thus, abusive use of a single antibiotic could result in resistance to an array of different antimicrobials (Pal et al. 2015). Stability and increase in resistance usually occur by a combination of both new mutations and effective horizontal gene transfer among bacterial species. Potential risks intensify, as pathogenic bacteria can pick up resistant determinants from non-pathogenic bacteria (Van et al. 2019). The prolonged and increased use of antimicrobials in animal husbandry in the SSA significantly contributes to the global emergence and spread of AMR microbes in the food chain. Therefore, successfully mitigating the menace of AMR in SSA would require giving special consideration to the role(s) of food animals as principal vectors in the emergence, maintenance, and circulation of superbugs from farm to fork.

A major challenge in the public health sector over the last decade has been the emergence of multidrug-resistant (MDR) pathogens (Santajit and Indrawattana 2016). Upsurges in the incidence of extended-spectrum beta-lactamase (ESBL), New Delhi metallo-beta-lactamase 1 (NDM1), and mobile colistin-resistant (mcr) in FAs among other genes posit that more stringent actions be employed in animal production processes (Ghafur et al. 2019; Perez-Rodriguez and Mercanoglu 2019). These previously less common MDR pathogens in the food industry now present a different epidemiological trend. Reports from different researchers around the globe have indicated their significantly high prevalence in FAs and animal-derived foods; and their increase has been linked to the imprudent use of antibiotics in FAs (Dulo et al. 2015; Rasmussen et al. 2015; Djeffal et al. 2017; Seni et al. 2017; Kagambega et al. 2018; Perez-Rodriguez and Mercanoglu 2019). These reports debunk the general assent of researchers attributing the sole transmission of MDR to be from person to person; hence, FAs could also be implicated in the transmission chain of MDR pathogens, and not merely limited to nosocomial spread (CDC 2018). Data supporting the upsurge of AMR in food animals showed that *Salmonella* isolates obtained from poultry birds in Algeria displayed a higher resistance (51.11% and 26.6%) to ciprofloxacin and cefotaxime than strains from humans (14.5% and 16.2%) (Djeffal et al. 2017). The production of ESBLs identified as *blaCTX-M-15*, *blaTEM*, and *blaCTX-M-1* was found among 12 and 8 poultry and human isolates, respectively. A multi-locus sequence typing (MLST) and clustering carried out suggested the isolates from the two sources (humans and birds) to be of common origin. Similarly, in another study in Burkina Faso, all human and poultry-borne Typhimurium serovars of *Salmonella enterica* showed resistance to 5 commonly used antibiotics (streptomycin, ampicillin, chloramphenicol, sulfonamide, and trimethoprim) in the country (Kagambega et al. 2018). The *strA*, *strB*, and *aadA1*, *blaTEM-1B*, *catA1*, *sul1 and sul2*, and *dfrA1* genes were linked to the resistance observed against the tested antibiotics. Furthermore, MLST also revealed genetic similarity between the isolates obtained from human and poultry.

## Food safety concerns associated with antimicrobial use in food animal production

**Fig. 3:**
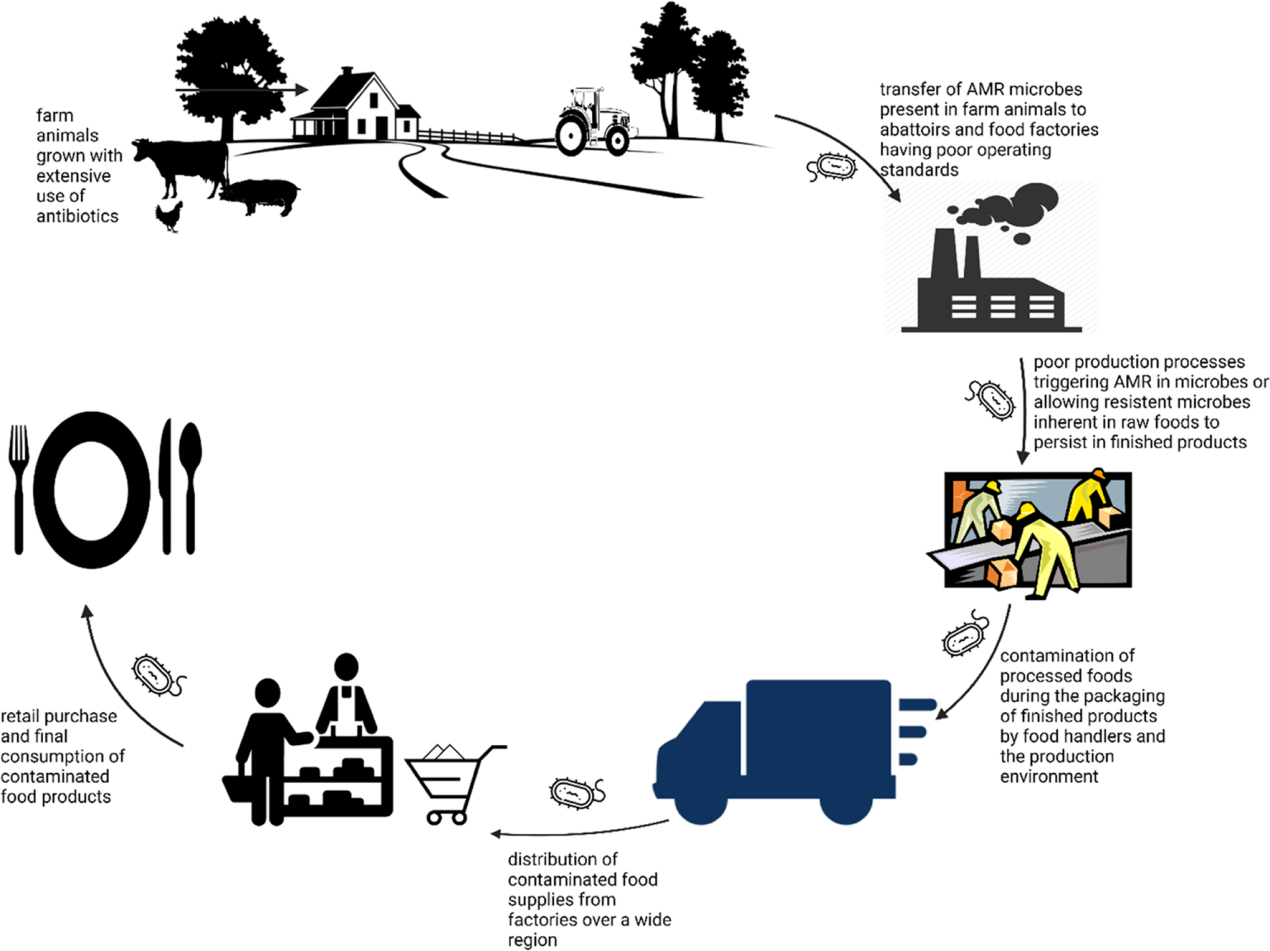
Farm-to-fork transmission of AMR

The food production chain is integral in the effective transmission of AMR pathogens. This claim holds relevance because nutrient-packed foods are not sterile, and often contain inherent microorganisms from the food source or those that are introduced as contaminants along the processing line via recontamination or cross-contamination (Cahill et al. 2017). The transmission of antimicrobial-resistant pathogens across the food production chain is not only limited to animal-based foods but also plant foods, because food crops may be contaminated by untreated effluent wastes and manures from farms with AMR pathogens (Perez-Rodriguez and Mercanoglu 2019; Xu et al. 2022). Although substantiation of the clear-cut mechanism for the transmission of antimicrobial-resistant pathogens from the food chain down to the consumer remains in progress, it is however imperative to admit the possibility of antimicrobial-resistant pathogens gaining entry and persisting right from the onset of production from the raw materials, through food processing techniques and the environment (Varraes et al. 2013; Founou et al. 2021) (Fig. 3).

AMR traits may still be successfully transmitted along the food chain, even though processing methods attain total elimination of microbial populations. This is because genetic material released from the destroyed microbe may be picked up through transformation from pathogens or commensals present in the gut of the consumer (Horn and Bhunia 2018). A consequent rapid decline in demand for animal-derived foods and loss of consumer confidence in product safety and quality may ensue following widespread information about the possibility of hazards of zoonotic AMR and MDR pathogens present in the foods. Hence, the rise in the incidence of MDR pathogens in FAs may not only increase global food safety concerns but could also translate to considerable but avoidable financial losses (Perez-Rodriguez and Mercanoglu 2019). Findings from North Central Nigeria indicated a high concentration of antimicrobial residues present in fish samples, and this has raised concerns about food safety and quality within the region (Alhaji et al. 2021). Consumption of such products can result in some public health challenges, ranging from disruption of the gut’s microbiota to the trigger of certain allergic reactions, and resistance in certain microbes. A spillover effect of this could result in a negative impact on the international trade of agro-products (Alhaji et al. 2021). The loss of consumer confidence in food quality may as well extend to plant-based foods, as sewage-contaminated irrigation systems and manures employed in growing food crops may harbor AMR genes or foodborne pathogens. (Durso and Cook 2014).

Documented scientific reports have demonstrated strong links between antimicrobial-resistant pathogens in FAs and the use of antimicrobials in animal husbandry (ECDC/EMEA 2009). In a study in Ethiopia, raw meat purchased from urban markets was reported to have a prevalence of about 26% of *E. coli* (Messele et al. 2017)*.* The isolates were more frequently found in chicken, followed by mutton, chevon, and beef. While 95% of the isolates displayed some level of resistance to the tested antimicrobials, 46% showed multidrug resistance (MDR) to at least three antibiotics. The *tet(A)*, *blaCMY*, and *suIII* genes encoding resistance for tetracycline, beta-lactams, and sulfonamides were reported abundantly in the isolates (Messele et al. 2017). Another recent study also confirmed the observable increase of AMR pathogens in animal-based foods. In the study on MDR *E. coli* from raw meat and cloacal swab samples obtained from abattoirs in Tanzania, 55.2% of the samples were *E. coli* positive, out of which 69.3% exhibited resistance to multiple agents (Mgaya et al. 2021). AMR towards tetracycline (91.9%) was the highest, and this correlates to the high rate of tetracycline use in FAs production in Tanzania as earlier discussed (Mgaya et al. 2021). Furthermore, sulfamethoxazole-trimethoprim (80.5%) and ampicillin (70.9%) followed next in the resistance profiling, while 8.6% resistance to imipenem was reported, and 4.7% of the isolates were ESBL-producing *E*. *coli*. The *CTX-M* gene encoding resistance to beta-lactams was present in two positive isolates, while the *qnrS* gene for quinolones resistance was present in eight samples. These reports have provided strong evidence indicating potential consumer risk as regards animal-based food (Mgaya et al. 2021).

## Regional drivers of antimicrobial use in food animal production in SSA

Several studies have demonstrated the use of antimicrobials in food animal production in SSA (Alhaji et al. 2018; Adebowale et al. 2020; Kimera et al. 2020a; Mupfunya et al. 2020; Paintsil et al. 2021; Mankhomwa et al. 2022). This practice among farmers is driven by some common factors that mount undue pressure on them to either continuously abuse or overuse antimicrobials, especially for growth promotion. Briefly, most farmers in SSA region had recourse for antibiotics use primarily for the following reasons:

1. Proper veterinary services are expensive and inaccessible, leaving farmers to resort to advice from other farmers, family, and friends.
2. Antimicrobial drugs are available and easily accessible without restrictions. They could easily be accessed as over-the-counter drugs without veterinary prescriptions.
3. The “positive” outcome from previous use of antibiotics endorses a repeat when the same challenges are encountered.
4. Deterioration of animal health necessitates a rapid response.
5. AMU compensates for poor hygiene and production when administered prophylactically.
6. AMU increases profitability as it reduces mortality and increases the total mass yield of the animals.
7. The poor educational status and level of literacy of the farmers regarding proper AMU and AMR.

A study in Tanzania demonstrated that 92% of the surveyed farmers lacked proper knowledge of management systems for the prevention and control of most diseases and thus depend on prophylaxis (Kimera et al. 2020a). About 95% complained about inadequate veterinary extension services, while over 65% enjoyed profit increases from a shortened period of farming as antimicrobial use as growth promoters boosted their animal yield.

From a study done in Blantyre, Malawi, among intensive small-scale farmers, it was reported that (i) the cost of practicing and maintaining proper biosecurity measures, (ii) the need to compete favorably with large-scale farmers in making profits, and (iii) the cost of veterinary services were the foremost drivers for the use of antibiotics in food animals within the region (Mankhomwa et al. 2022). In addition to this, regular human antibiotics are readily available over the counter and relatively cheaper thereby becoming more accessible and affordable for farmers to use for the treatment of some animal diseases. Also in North Central Nigeria, socioeconomic factors, intensive management, poor biosecurity measures, and hygiene have been identified as the foremost drivers of antimicrobial misuse leading to AMR (Alhaji et al. 2021).

## Impact of antimicrobial use in food animal production in SSA

### Public health impact

The indiscriminate and prolonged use of antimicrobials in FAs provides conducive systems that drive the development and subsequent spread of antimicrobial-resistant pathogens. Animal health may or not be affected by drug-resistant zoonotic pathogens, but this subsequently affects human health significantly, as the pathogens get transmitted to the human population directly or indirectly through water, food, or the environment via leached animal waste and manure (Ma et al. 2021). Irrefutable scientific observations, with clear evidence, have linked myriads of AMR bacteria as well as resistant genes to animal-derived foods at the different phases of processing (Atterby et al. 2019; Kim et al. 2019; Wolfe 2023). In a study carried out in Cambodia, the presence of the same *mcr-1/2* gene and ESBL genes was reported in both FAs (swine and poultry) and the farmers rearing them (Atterby et al. 2019). In a similar study, a strong correlation was made between the presence of *mec-A* gene encoding for methicillin resistance in *S. aureus* in dairy farm personnel and the intensity of contact with animals in Greece (Papadopoulos et al. 2019).

The transfer of antimicrobial-resistant pathogens to humans accounts for several health problems. AMR bacteria usually account for high morbidity, presenting chronic illnesses with increased recurrence rates, and high chances of disease metastasis ultimately reducing the quality of life and terminally resulting in death for a number of cases (Buzón-Durán et al. 2017; Alhaji et al. 2021). Quantifying the exact effect of AMR and its associated infections in terms of morbidity and mortality may be difficult since secondary resistance may be muddled up with initial infections. However, it is believed that infections due to AMR could aggravate disease symptoms and impair treatment management, thus increasing the cost of treatment, requiring longer hospitalization, and eventually could result in the death of patients in some cases (Capita and Alonso-Calleja 2013; Buzón-Durán et al. 2017). Greater concerns have been raised about the spread of resistant pathogens to the immunosuppressed human population, such as persons living with HIV, malnourished children, the elderly, and cancer patients (ECDC/EMEA 2009).

Other rising public health concerns following the abusive use of antibiotics in FAs are linked to drug residue in animal products. This usually ensues due to the lack of adherence to safe withdrawal periods in treated animals (Mdegela et al. 2021). Studies have shown that some of these antibiotics are only poorly denatured by cooking, as a good fraction remains chemically active and measurable even after cooking (Javadi 2011). These rather small quantities of antibiotics present in food can adversely affect the consumer’s health. Exposure to certain beta-lactam antibiotics, aminoglycoside, sulfonamides, and tetracycline may result in allergies among users who have been previously sensitized (Darwish et al. 2013). Liver injury following certain allergic reactions from metabolite-modified hepatic cells, gastrointestinal disturbances, and teeth yellowing in some cases can be traced to exposure to antibiotics from food (Darwish et al. 2013; Moyane et al. 2013). Persistent consumption of animal-derived products with residues of antibiotics can over time confer resistance to the normal microflora of the gut by developing tolerance to them, even when the products are void of resistant bacteria. Moreover, some commensal microorganisms could in some instances become opportunistic pathogens causing severe and life-threatening diseases in immunocompromised individuals.

During food processing, the alteration of the fermentation of some animal products (e.g., milk and meat) due to the presence of drug residues can pose a significant threat to public health. This is possible because some commonly used industrial fermenters may be susceptible to the residual antibiotics in the raw materials, hence interfering with and altering the fermentation process. For example, previous studies reported how an altered fermentation process resulted in the outbreaks of foodborne illnesses as pathogens present in food persisted following poor fermentation (Darwish et al. 2013; Moyane et al. 2013).

### Environmental impact

Animal wastes collected from livestock production are typically channeled by most farmers into the production of manure and compost for agricultural benefits. However, this eco-friendly means of enriching the soil also introduces an array of pathogens including superbugs to the surrounding environment and water bodies (Gentry-Shields et al. 2015; Hu et al. 2017; Fang et al. 2023). In north-western Nigeria, most poultry farmers utilize the fecal matter from their farms as manure and this often serves as a major source of contamination, possibly contributing to the release of AMR into the environment (Alhaji et al. 2018). Soil surfaces and groundwater easily become contaminated with pathogenic microbes through the application of animal wastes or manure as (bio)fertilizers. It has been observed that after the application of animal waste/manure, pathogens contained in manure could survive for extended periods, with bacteria reported to survive for about 2 months (McLaughlin et al. 2012; Hu et al. 2017). Both the safety and quality of water resources become compromised as the levels of manure-borne pathogens begin to increase in aquatic bodies. The manure-borne pathogens can potentially impact aquatic organisms negatively (Hu et al. 2017). Moreover, most rural dwellers in SSA resort to using contaminated water from rivers/streams for drinking and other domestic use.

A 12-year study in Tanzania found that specific strains of ESBL-producing *E. coli* (ST2852, ST131, and ST38) intersect human, animal, and their environments (Seni et al. 2017). Although a variable occurrence was observed, the identification of the *blaCTX-M-15* allele in the *IncF* conjugative plasmids points to the fact that possible transmission across the three entities exists. In a similar study in Angola, there was an unusually high frequency of the blaCTX-M-15 gene across enterobacteria from diverse non-clinical niches which included healthy humans, farm animals, wastewater, and rivers (Ribiero et al. 2016). The widespread occurrence of the blaCTX-M-15 gene across different niches is suggestive of its tendency to be readily acquired and mobilized by bacteria of different genetic backgrounds. With environmental microbes implicated in the process, reservoir organisms for AMR become increasingly high (Ribiero et al. 2016).

Although the concentration of residual antimicrobials present in animal manure is not significantly high, they still impact the microbial communities in the environment, as soil-bound antibiotics possess potent bioactivity (Peng et al. 2014). This changes the phylogenetic structure of the soil, with a consequent expansion of resistance and distress within the micro-ecosystem (Hu et al. 2017). A spillover of this is seen in the alteration of biomass production and biotransformation of elements as well as the buildup of organic pollutants in surrounding soil and water bodies. It has also been reported that the physiological and developmental processes of certain plants could be affected by antimicrobial agents from the environment (Barkitova et al. 2016; Fang et al. 2023).

### Economic concerns

The intensive use of antibiotics in animal production seems to improve yield and save production costs at a peripheral glance. This is because it improves animal mass and feed conversion, saves costs, and compensates for poor production and biosecurity practices (Patel et al. 2020). It also cuts down animal mortality rate and reduces the net microbial load present on raw food samples, rendering them easier to process and safer for consumption (Van et al. 2019). However, a bigger picture reveals the impact of prolonged indiscriminate use of antimicrobials, as it influences the increase in AMR. This consequently takes a negative and more detrimental toll on the global economy (Landers et al. 2012; Patel et al. 2020).

The surge in AMR in pathogens has brought about an increased burden on healthcare systems and serious financial stress on society due to the high costs of managing them. Infections due to antibiotic-resistant pathogens usually show more severe clinical outcomes accompanied by exacerbated illnesses requiring prolonged in-hospital treatment, when compared to infections caused by antibiotic-susceptible pathogens (Aidara-Kane et al. 2018; Alhaji et al. 2021). For example in the mid-1990s when the cost of managing diseases due to resistant pathogens started escalating, over 4 billion dollars was expended in the USA in the treatment of infections attributed to antibiotic-resistant pathogens (Capita and Alonso-Calleja 2013). The European Centre for Disease Prevention and Control/European Medicines Agency (ECDC/EMEA) also gave estimates of about 1.3–2.7 billion and 1.5 billion dollars spent in the USA and EU, respectively, as associated annual costs of healthcare related to AMR, though an underestimation by most studies was asserted (ECDC/EMEA 2009).

A more recent estimate by the Centers for Disease Control and Prevention put the annual economic loss due to AMR-associated infections at a whooping sum of 28 billion dollars in the USA, with an extra 12.4 billion dollars in productivity loss (CDC 2018). For lower and middle-income countries, especially Sub-Saharan Africa, the disease burden due to the AMR is expected to be higher, although there is a dearth of realistic financial estimates to substantiate it due to the lack of an effective disease reporting and surveillance system (Madina et al. 2020).

Global projections for the next few decades predicted a cumulative cost of 100 trillion dollars in economic loss due to AMR and an estimated 10 million individuals at risk of diseases due to AMR pathogens by 2050 (O’Neill 2016; Madina et al. 2020). This is expected to cause about 28.3 million increments in the total population of people living in extreme poverty such as the SSA, as the negatively impacted global GDP faces an annual decline of about 1.1–3.8%. Finally, an annual increase of between 300 billion and 1 trillion dollars is estimated to be committed to healthcare costs if the impact of AMR-associated infections gets aggravated (World Bank Group 2017).

## Control and mitigation of AMR in food animal production in SSA

Even though there is yet a directly proportional relationship between AMU and AMR in food animal production in SSA, AMU remains an indispensable determinant of AMR within the region. As the emergence of pan-resistant microorganisms that evade all commercially available antibiotics continues, tying all loose ends contributing to the surge and spread of AMR becomes imperative. Therefore, the necessity for proper control and mitigation of the uprising, selection, and dissemination of antimicrobial resistance in microorganisms in FAs cannot be overstated (Capita and Alonso-Calleja 2013; Perez-Rodriguez and Mercanoglu 2019). We discuss major aspects that can be considered for the sustainable control and mitigation of AMR driven by antibiotic use in food animals in SSA.

### Regulation and control of AMU in food animal production

One of the factors driving the development, maintenance, and spread of AMR in microorganisms among FAs includes the indiscriminate use of in-fed antibiotics for the enhancement of animal growth (Hu et al. 2017). The selective pressure on bacterial species induced by the misuse of antimicrobials can be effectively reduced in FAs by proper control of the consumption of antimicrobials, consequently reducing the prevalence of resistant bacteria. Supporting data demonstrated that the proportion of tetracycline-resistant bacteria in humans and swine experienced a decline, following the ban of tetracycline as an additive for growth promotion in feeds within the EU during the mid-1970s (Ma et al. 2021). There was also a rapid decline from about 80 to 5% in the incidence of vancomycin-resistant Enterococci in Denmark, which correlated to the ban on avoparcin in 1995 (Ma et al. 2021). Conversely, the prevalence of vancomycin-resistant *E. faecium* had a 43% increase from its basal 27% in the mid-1990s, following increased use of virginiamycin in Danish broilers. The findings of Björkman et al. (2021) in a qualitative study, conducted based on the One Health Initiative, showed a correlation between the cooperation of farmers/producers with policymakers and the resultant success of the ban on antibiotics in animal production, especially those employed for non-therapeutic purposes in Sweden. Both parties demonstrated proper education on the impact of AMU in the progression and spread of AMR. Their study showed that both veterinarians and farmers focused on prevention, biosecurity, and animal welfare in production methods rather than the use of antibiotics while policymakers provided some financial support to achieve the goal (Björkman et al. 2021). This was all geared toward the fight against antibiotic resistance (ABR) in Sweden. Quelling the non-therapeutic use of antimicrobials, especially for growth promotion in FAs, can effectively mitigate the spread of AMR across humans, animals, and our environment (Hu et al. 2017).

Although all national and global policies aimed at containing AMR recommend the restrictive use of antimicrobial agents, only the practical application of these theoretical concepts would translate to the needed change, especially in the SSA region (Kimera et al. 2020a). Many may interpret this to mean regulations on the withdrawal period before harvesting eggs (from poultry) or milk from treated animals or even slaughtering for meat, but it extends beyond that. The much-needed action is a total cut down on all non-therapeutic use of antibiotics, as scientific research bears compelling evidence implicating the misuse of antibiotics in FAs and the development of resistance (Hoelzer et al. 2017; Aidara-Kane et al. 2018; Björkman et al. 2021). The adoption of these policies and protocols enacted in developed countries may be difficult in the developing world due to certain regional peculiarities and challenges. Nevertheless, this can be achieved by creating strong or strengthening existing legislation, proper monitoring and evaluation of farm practices, and integrating protocols that take into consideration the pressure on farmers for the use of antibiotics.

### Advocating for the use of alternatives to antibiotics in food animal production

Enhanced and optimized biosecurity practices, good hygiene, and good farming practices have great potential in reducing the risk of disease outbreaks in livestock production. However, applying those measures does not rule out the chances of disease occurrence, as serious consequences may ensue in animal production devoid of antibiotics use (Cevantes 2015; Reuben et al. 2021b). Gut colonization with pathogens, poor animal mass yield, malabsorption of nutrients, increased contamination of the environment with entomopathogens, and increased public health risk from zoonotic diseases are major upsurges that may arise from the practice (Reuben et al. 2021b). Advocating for the use of safe and sustainable alternatives to antibiotics, especially as growth promoters and prophylaxis may significantly mitigate and reduce the emergence and spread of FA-associated antibiotic-resistant bacteria in SSA. Some of the antibiotic alternatives that can be explored include probiotics, prebiotics, postbiotics, bacteriophage therapy, vaccination, endolysins, bacteriocins, antimicrobial peptides, and phytobiotics, which have shown credible outcomes in their use and are continually being researched on.

i. ***Prebiotics***

Prebiotics are substrates or feed ingredients that are selectively utilized in the intestinal tract yielding beneficial impact in their host by stimulating specific indigenous gut microbes, enhancing their growth and composition, and consequently inhibiting pathogenic bacteria (Cheng et al. 2014; Gibson et al. 2017; Reuben et al. 2021b). Initially identified as non-digestible food ingredients, they primarily consist of non-starch polysaccharides and non-digestible oligosaccharides, like mannan and fructooligosaccharides and inulin. Currently, their scope has expanded to include protein hydrolysates, polyunsaturated fatty acids convertible to conjugated fatty acids, polyols, and other plant extracts (Gibson et al. 2004; Cheng et al. 2014; Rickie 2015; Reuben et al. 2021b). The practical use of prebiotics started in the mid-1980s when they were used as feed additives. In current practice, some of the best prebiotics include multifunctional oligosaccharides and acidifiers (Cheng et al. 2014; Solis-Cruz et al. 2019).

An array of mechanisms govern the function of prebiotics, most of which have been traced to a consortium of bacterial microbiota in the gut of the host. Even though the exact mechanisms that outline the process through which prebiotics exert their beneficial effects are yet to be fully elucidated, intricate interactions between microflora balance, gut morphology, and host physiology control these processes. These interactions stimulate the immune system, inhibit gut pathogens, enhance the digestibility of food substrates, and trigger other beneficial metabolic changes, thus promoting better performance and animal health (Ahmed et al. 2018; Solis-Cruz et al. 2019; Reuben et al. 2021b).

Although glaring benefits follow the use of prebiotics, some concerns have been raised. Incorporating large quantities of prebiotics into animal feeds may induce diarrhea, bloating, and other adverse reactions following fermentation in the gut. Also, the unclear connection between the physiological function and the structure of prebiotics affects their application within variable animal species, with chances of mutual antagonism occurring. Other concerns include the affordability of prebiotics, as high production costs may influence the prices of animal products (Cheng et al. 2014).

ii. ***Probiotics***

The definition of the word probiotics has undergone a series of evolution ever since its coinage. From its initial use in 1953 by Kollath to describe both organic and inorganic substances used to improve the health of malnourished patients (Kollath 1953), to Parker’s 1974 definition encompassing living organisms and other substances that promote microbial balance in the gut (Parker 1974), the term has evolved. Nowadays, most researchers describe probiotics as live cultures of microorganisms intentionally introduced to promote the health and well-being of the host (Reuben et al. 2020, 2021a, 2021b). The world health organization defines probiotics as microorganisms capable of conducing health benefits to their host upon live administration in the appropriate quantity (FAO and WHO 2006; Seighalani et al. 2023).

Probiotics have been observed to confer several health and nutritional benefits to food animals. They promote animal development and maturation (Nielmialtowski et al. 2005), and also enhance feed intake, digestibility, and efficiency (Arowolo and He 2018; Rehman et al. 2020). Other benefits include the enhancement of health performance and immune response (Al-Sagheer et al. 2019), egg production and quality in poultry (Xiang et al. 2019), meat yield and quality (Soomro et al. 2019; Reuben et al. 2021a, 2022), and milk composition and production for ruminants (Reuben et al. 2021b). These benefits are achieved through several defined mechanisms including competitive colonization and exclusion of pathogens, production of bacteriocins and other antimicrobials peptides and organic acids, and stimulation of gut immune responses, altering the expression of virulence in pathogens via cell-to-cell interactions (Cheng et al. 2014; Reuben et al. 2021b).

With increased advances in probiotic research, novel and more effective probiotic strains are being discovered, and existing probiotic activities are being optimized and put to better use. For an organism to be utilized as a probiotic, it is of necessity that it possesses certain characteristics, and as such, it must be toxin free and nonpathogenic, achieving the status of GRAS, generally regarded as safe, confer benefits to the host, have tolerance for bile salts and acids within the host’s gut, possess antagonistic activities against pathogens, capable of adhesion and competitive colonization of the host while resisting removal from the gut due to intestinal contraction, and remain viable during the processing, packaging, and storage processes (Ezema 2013; Gibson et al. 2017; Reuben et al. 2021b).

Globally, different commercial probiotic products are marketed and they can easily be purchased and used by FA producers in SSA. Some of these commercially available probiotic products and those at various phases of trials include *Lactococcus lactis* subsp. *lactis* CV56 (Yang et al. 2013), *Enterococcus durans* (Fijan 2014), *Enterococcus* (*E. faecium*) (Cheng et al. 2014; Reuben et al. 2021a), *Pediococcus* (*P. acidilactici*), *Lactobacillus* (*L. plantarum*, *L. acidophilus*, *L. farciminis*, *L. rhamnosus*, *L. casei delbrueckii* subsp. *bulgaricus* (Fijan 2014; Latorre et al. 2016; Reuben et al. 2021a)*, Bifidobacterium* (Reuben et al. 2021a), *Bacillus* (*B*. *licheniformis*, *B. cereus var. toyoi*, *B. subtilis, B. coagulans, B. subtilis)* (Newberry 2012; Reuben et al. 2021a; Zokaeifar et al. 2014)*, Streptococcus* (*S. infantarius*, *S. thermophilus*) (Fijan 2014; Reuben et al. 2022), *Bacteroides* (Latorre et al. 2016; Reuben et al. 2021a), *Escherichia coli* Nissle 1917 (Fijan 2014), yeast (*Saccharomyces cerevisiae*, *S. boulardii, Candida*, and *Kluyveromyces*), *Aspergillus*, and *Trichoderma* (McFarland 2007; Vieco-Saiz et al. 2019; Reuben et al. 2021a)

iii. ***Antimicrobial peptides, bacteriocins, and postbiotics***

Emerging concerns following the use of probiotics are centered on the viability of the probiotic organism. Also, virulent and antimicrobial resistance genes could be spread through probiotic organisms to other gut commensals via horizontal gene transfer. Other concerns include problems of undesirable inflammatory responses and incidents of septicemia and bacteremia (Kothari et al. 2019). Notwithstanding, the achievement of desired health benefits is not necessarily dependent on the viability of probiotic strains, as shown by compelling evidence (Reuben et al. 2021a, 2022). Nonviable whole-cell, purified cell-wall, and other components of probiotic organisms still confer similar benefits (Raman et al. 2016). These findings back the development of postbiotics; food additives consist of nonviable components and or purified extracts from selected organisms conferring health benefits on use (Rad et al. 2020; Reuben et al. 2021b).

Postbiotics usually carry out their effect by employing immunologic modulations and activations, countering the effect of pathogenic bacteria and reducing inflammation in the gut. They also exhibit antagonistic activities on pathogenic bacteria through the release of bacteriocins and antibacterial peptides, both of which are biochemical substances composed mainly of proteins and are secreted by a specific strain of microbe having antimicrobial activity against microbes of different strains or unrelated strains; examples include enterocin, pediocin, lactococcin A, etc. (Reuben et al. 2019, 2020). Other benefits include antioxidant and hepatoprotective properties (Compare et al. 2017; Dinic et al. 2017; Aguilar-Toala et al. 2018; Reuben et al. 2021b). However, the beneficial effects of postbiotics are hinged on the type of metabolites secreted and the microbial strain used (Compare et al. 2017).

iv. ***Vaccines***

The use of vaccination alongside the regulated use of antimicrobials in animal production, implementation of proper biosecurity measures, and good hygiene practices have been observed to positively impact animal production (Chalier et al. 2022). The use of vaccines helps confer protection to the animals from disease pathogens and drastically reduces the use of antimicrobials in production. This comes to light as current strategies for improving animal health are now channeled to animal resilience, disease prevention, and advanced monitoring for rapid detection and intervention (Chalier et al. 2022).

In a study conducted in South Africa, chickens vaccinated with attenuated live vaccines (ts-11 and 6/85MG) were challenged with virulent field *Mycoplasma gallisepticum* (MG) and QX-like infectious bronchitis virus (IBV) strains (Bwala et al. 2018). Although they have varied capacities to inhibit MG replication in different tissues, both vaccines offered nonspecific protection against IBV. There are other useful data on vaccine efficacy in reducing disease severity and in most cases inhibiting the development of disease symptoms in the vaccinated birds (Bwala et al. 2018). In another study, investigating the effectiveness of the *S. aureus* vaccine formulated with liposomes and ODN-CpG against natural *S. aureus* inflammatory infections in both young and matured cows, findings revealed a 67% reduction in the development of new *S. aureus* inflammatory infection (IMI) in the vaccinated group as compared to the control group (Camussone et al. 2022).

Programs directed at the detection, prevention, and control of *Salmonella* in layer flocks in farms producing eggs have been enacted in the European Union. Among other biosecurity measures implemented in the member states is the vaccination of laying hens against *Salmonella enteritidis* which must be observed in poultry farms that are unable to achieve less than 10% prevalence. Since its implementation in 2008, there has been a plausible decline in the cases of *Salmonella* in laying hens, with a consequent drop in the number of cases of human foodborne infections within that region (Capita and Alonso-Calleja 2013).

## Conclusion

The discovery and use of antimicrobials have been a major contribution to the advancement of medicine, veterinary medicine, and animal husbandry, thus enabling the production of healthier and stronger animals. However, the development of antimicrobial resistance in microbes, though a usually slow and gradual process, appears to be escalating throughout the world, especially the SSA. These negative outcomes have been linked to the imprudent use of antibiotics among humans, animals, and food production systems.

In this review, we highlighted the overall regional drivers as well as the public health, environmental, and economic impact of antimicrobial use in the production of food animals. Antimicrobial use is likely to increase with even exacerbated outcomes unless cost-effective, safe, and sustainable alternatives to antibiotics, especially probiotics, prebiotics, bacteriocins, antimicrobial peptides, bacteriophages, vaccines, etc. are urgently advocated for and used in food animal production in SSA. These, in addition to the implementation of strong legislation on antimicrobial use, and improved hygiene will help mitigate the public health concerns associated with antimicrobial use in food animals and improve the well-being and safety of food animals and their products.

### Limitations

This review comprehensively assessed the current practices of antimicrobial use in food animal production and associated food safety concerns in SSA. While this review serves as a regional atlas of antimicrobial use in food animals and a valuable resource for future research, our assessment was limited by the paucity of requisite data and the absence of a unified surveillance, reporting, and data management system for AMR and AMU across the SSA. Our assessment was only limited to the studies that were published and available in the databases examined.

## Data Availability

All data supporting the findings of this study are available within the paper.
